# Automatic identification of the endangered hawksbill sea turtle behavior using deep learning and cross-species transfer learning

**DOI:** 10.1242/jeb.249232

**Published:** 2024-12-23

**Authors:** Lorène Jeantet, Kukhanya Zondo, Cyrielle Delvenne, Jordan Martin, Damien Chevallier, Emmanuel Dufourq

**Affiliations:** ^1^ African Institute for Mathematical Sciences, Muizenberg, Cape Town, 7945, South Africa; ^2^ African Institute for Mathematical Sciences, Research and Innovation Centre, KN3 Kigali, Rwanda; ^3^ Stellenbosch University, 7602, Stellenbosch, South Africa; ^4^ Unité de Recherche BOREA, MNHN, CNRS 8067, SU, IRD 207, UCN, UA, Station de Recherche Marine de Martinique, Quartier Degras, Petite Anse, 97217 Les Anses d'Arlet, Martinique, France

**Keywords:** Accelerometer, Behavioral classification, Bio-logging, Neural networks, Machine Learning, Animal behavior

## Abstract

The accelerometer, an onboard sensor, enables remote monitoring of animal posture and movement, allowing researchers to deduce behaviors. Despite the automated analysis capabilities provided by deep learning, data scarcity remains a challenge in ecology. We explored transfer learning to classify behaviors from acceleration data of critically endangered hawksbill sea turtles (*Eretmochelys imbricata*). Transfer learning reuses a model trained on one task from a large dataset to solve a related task. We applied this method using a model trained on green turtles (*Chelonia mydas*) and adapted it to identify hawksbill behaviors such as swimming, resting and feeding. We also compared this with a model trained on human activity data. The results showed an 8% and 4% F1-score improvement with transfer learning from green turtle and human datasets, respectively. Transfer learning allows researchers to adapt existing models to their study species, leveraging deep learning and expanding the use of accelerometers for wildlife monitoring.

## Teaser

**Summary:** Transfer learning is an effective tool for leveraging deep learning in acceleration-based behavior identification for wildlife monitoring, enabling existing models to be adapted to the species under study.

## INTRODUCTION

Monitoring animal behavior offers a non-invasive method for studying wildlife in their natural habitats, providing valuable insights into their ecology and physiology. Through behavioral analysis, researchers can deepen their understanding of how animals interact with their environment, elucidating key aspects of their energetic strategies, survival mechanisms and reproductive success (McHuron et al., 2018; Sansom et al., 2009; Zhang et al., 2004). Behavior monitoring also remains a critical tool for evaluating species’ adaptations to extreme conditions (Abernathy et al., 2019; Sergio et al., 2018) and rapid environmental changes (Beever et al., 2017; Buchholz et al., 2019). Furthermore, it enables the measurement of remarkable physiological capabilities, such as long-distance migration and deep breath-hold diving, facilitating a more comprehensive understanding of the mechanisms driving these behaviors (Boel et al., 2014; Fossette et al., 2010; Pedersen et al., 2018).

Bio-logging has emerged as a valuable tool for studying animal behavior, enabling continuous monitoring of animals in their natural habitats. It involves deploying on-board sensors that collect high-resolution data on animal behavior, physiology and environmental conditions. Among these sensors, the accelerometer is particularly effective for recording animal posture and movement. It operates as a piezoelectric sensor and translates the forces exerted on a mass into wave-like voltage signals. These forces include the gravitational force and the force of inertia induced by the posture and movement of the animal, respectively (Brown et al., 2013). By positioning three accelerometers orthogonally, researchers can identify behaviors characterized by specific postures and movements, such as locomotion, feeding and inactivity (Shepard et al., 2008).

Automating data processing is a key challenge in acceleration-based behavior identification, with the aim of facilitating the analysis of large time-series datasets. Recently, deep learning has emerged as a promising tool to automatically analyze acceleration data for identifying the behavior of wild animals (Aulsebrook et al., 2024; Jeantet et al., 2021; Otsuka et al., 2024; Zhang et al., 2019a). Deep learning algorithms, also known as neural networks, are computational models that process data through multiple layers, performing both linear and non-linear transformations. They can automatically detect complex, highly discriminative features and patterns in the data, making them capable of classifying acceleration signals into distinct behavioral categories. Deep learning, in particular, has been at the forefront of advancements in behavior identification using accelerometers in livestock monitoring and Human Activity Recognition (HAR) (Bao and Xie, 2022; Kumar et al., 2023). In livestock, many studies have developed deep learning models to track behaviors such as feeding, drinking, walking and resting, and for detecting lameness in cattle, sheep and pigs, aiming to monitor animal welfare and productivity (Kleanthous et al., 2022b; Mao et al., 2023). In humans, accelerometers are widely used to track physical activity, identifying behaviors such as running, walking, standing and sitting, and are also employed to detect falls, which is especially important for care of older people (Choi et al., 2022; Nunavath et al., 2021; Ramanujam et al., 2021). Conversely, deep learning is still underutilized in the field of ecology, with limited research dedicated to developing new methods to facilitate its use or enhance its performance.

The limited use of deep learning in ecology, compared with that in livestock monitoring or HAR, may be attributed to the additional challenges of acquiring large, representative and accurately labeled datasets in ecological research. Deep learning algorithms are generally large models with numerous successive operational layers. Each operation relies on real-valued numbers, known as weights, which are optimized, or fine-tuned, during the training phase using labeled data to tailor the model to the specific task. As the number of layers in the model increases, its ability to discriminate features improves, but this also necessitates a larger labeled dataset for effective training (He et al., 2016). In contrast, studying wild animals presents challenges in data collection because of the need to minimize disturbance, environmental constraints and limited access to endangered species, resulting in smaller datasets. Additionally, the need for simultaneous observation to create a labeled dataset further complicates the process, leading to even smaller training datasets.

One of the primary risks of using a dataset that is too small for training deep learning models is overfitting. Overfitting occurs when the model's weights become overly tailored to the specific examples in the training data. Consequently, the model performs well on the training dataset but poorly on new, unseen data. To address data scarcity in other fields of ecology, researchers have turned to transfer learning techniques across a range of ecological tasks, including bioacoustics, camera traps, drone surveys and animal pose estimation from video recordings (the last three falling under computer vision) (Dufourq et al., 2022; Gray et al., 2019; Liao et al., 2023; Lu et al., 2021; Schneider et al., 2018). Transfer learning is a technique in which a model trained on one task is adapted and fine-tuned to a different, but related task. This method enables the use of deep learning models, with numerous layers, that have been pre-trained on large datasets, while mitigating the risk of overfitting on smaller, more limited datasets. For example, in bioacoustics, Batist et al. (2024) employed a pre-trained model, originally designed for classifying object categories from color images and trained on ImageNet, a dataset comprising over 15 million annotated images, to identify sounds emitted by black-and-white ruffed lemurs (*Varecia variegata*) using spectrograms, the visual representations of sound. Even though the datasets and tasks are not identical (classifying images versus classifying spectrograms), a neural network pre-trained on the image dataset can learn abstract patterns that are transferrable to the spectrogram dataset. While transfer learning has recently been utilized in acceleration-based behavior identification studies of livestock (Bloch et al., 2023; Kleanthous et al., 2022a), and has long been employed in the study of human behavior (Cook et al., 2013; Hernandez et al., 2020), it has not yet been applied to the study of wild animals.

The issue of data scarcity to train deep learning algorithms in ecology is especially notable for marine species. In this study, we explored the use of transfer learning to study hawksbill sea turtles (*Eretmochelys imbricata*). Among the six endangered sea turtle species, the hawksbill turtle is the most endangered along with the Kemp's ridley turtle (*Lepidochelys kempii*), both classified as critically endangered (IUCN, 2024). Martinique is an island in the Caribbean where a population of hawksbill turtles and juvenile green turtles (*Chelonia mydas*) reside (Lelong et al., 2024; Nivière et al., 2018). Understanding their behaviors is essential to elucidate how these endangered species utilize resources and adapt their energy strategies in a changing environment (e.g. rising water temperatures, coral bleaching and eutrophication; Li and Reidenbach, 2014), as well as under significant anthropogenic pressures (e.g. accidental bycatch in fisheries, disturbances related to tourism; Hayes et al., 2017; Louis-Jean et al., 2009; Siegwalt et al., 2022). Accelerometers are an effective tool for studying behaviors such as swimming, resting, breathing and feeding in marine turtles, thereby facilitating a deeper investigation into their energy strategies (Fossette et al., 2012; Jeantet et al., 2020a).

The green turtle population in Martinique is subject to significant monitoring (Bonola et al., 2019; Charrier et al., 2022; Siegwalt et al., 2022, 2020), and studies utilizing accelerometers have been conducted to automatically monitor their behaviors (Jeantet et al., 2020a, 2021). Conversely, studying the population of hawksbill turtles presents greater challenges, as individuals are more elusive than green turtles, making it difficult to equip them with bio-loggers. Furthermore, very few studies have employed accelerometers to investigate hawksbill turtles; to our knowledge, only two studies have explored the use of this technology to study their behavior (Jeantet et al., 2018; Okuyama et al., 2012). Consequently, there is a substantial lack of data and studies on this species, and the challenges in data collection make it difficult to apply deep learning algorithms to study their behaviors.

The aim of this study was to investigate the application of transfer learning for the automatic identification of behaviors in endangered species using accelerometer data, addressing the common challenge of small datasets in ecological research when training deep learning algorithms. We focused on a particularly challenging species to monitor, the hawksbill sea turtle, for which limited research and data exist. Firstly, we explored the use of a pre-trained model on a closely related species, the green turtle, whose postures and movement patterns are supposed to be similar to those of the hawksbill turtle in the expression of behaviors of interest, such as resting, feeding, swimming and breathing. For this species, a publicly available dataset and trained model exist. Secondly, we assessed the feasibility of applying transfer learning from a species with a noticeably different morphology from the hawksbill turtle, for which behavioral identification should rely on different features to those used for sea turtles. This approach enabled us to test whether the method is species specific or can be applied to a broader range of species, with minimal dependence between the species used in the pre-trained model and the species being studied. For this, we focused on humans, as data collection is more accessible and numerous datasets and pre-trained models are available. This study is the first to explore transfer learning for monitoring wild animals, aiming to propose a method that can be generalized across a broader range of species, thereby facilitating the wider application of deep learning in the study of animal behavior.

## MATERIALS AND METHODS

### Hawksbill dataset

#### Ethical note

This study meets the legal requirements of the countries in which the work was carried out and follows all institutional guidelines. The protocol was approved by the ‘Conseil National de la Protection de la Nature’ (http://www.avis-biodiversite.developpement-durable.gouv.fr/bienvenue-sur-le-site-du-cnpn-et-du-cnb-a1.html), and the French Ministry for Ecology, Sustainable Development and Energy (permit no. 2013154-0037), which acts as an ethics committee in Martinique. The fieldwork was carried out in strict accordance with the recommendations of the Prefecture of Martinique in order to minimize the disturbance of animals (authorization no. 201710-0005).

#### Data collection

**Fig. 1.**
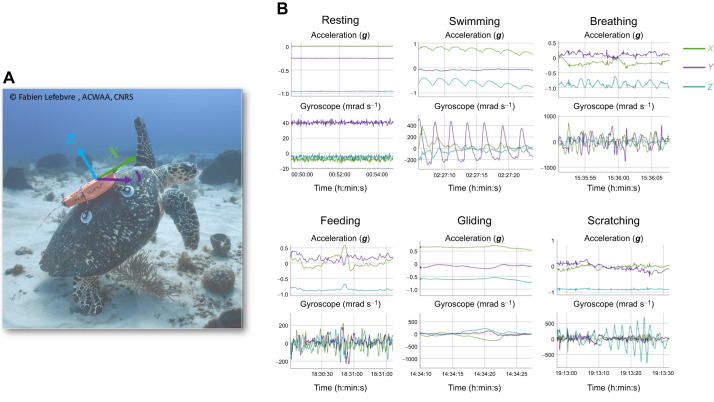
**On-board camera combined with a tri-axial accelerometer and tri-axial gyroscope deployed on a hawksbill sea turtle in Martinique.** The three arrows indicate the orientation of the accelerometer and gyroscope axes: *X* represents the front-to-back axis, *Y* the lateral axis, and *Z* the top-to-bottom axis (A). The plots on the right show the raw acceleration and gyroscope signals corresponding to each observed behavior of the hawksbill sea turtle (B). Photo credit: Fabien Lefebvre, ACWAA, CNRS.

The hawksbill turtle dataset was collected through fieldwork conducted in Martinique, Caribbean Island, France, from November 2022 to May 2023. Six free-ranging hawksbill turtles, *Eretmochelys imbricata* (Linnaeus 1766), were manually caught, measured and identified as described in Nivière et al. (2018) and Jeantet et al. (2020a). We equipped them with CATS (Customized Animal Tracking Solutions) devices over a 2 day period using four suction cups attached to their carapace and an automatic release system (Fig. 1; see Jeantet et al., 2020a, for details). Each CATS device comprised a video recorder combined with a tri-axial accelerometer, a tri-axial gyroscope and a pressure sensor. The devices were configured to continuously record acceleration and angular velocity (gyroscope) at a rate of 20 Hz and pressure at 1 Hz. The video recorders were programmed to record until nightfall (18:00 h) and resume at daybreak (06:00 h). The maximum battery capacity was considered to provide a recording capacity of 18 h of video footage and 48 h for the other sensors.

#### Video annotation and data preprocessing

Video recordings captured by the CATS devices were analyzed through visual inspection using the custom-written software TurtleCap (https://github.com/Vadym-Hadetskyi/TurtleCap) to identify behaviors. The corresponding start and end times of each observed behavior were annotated, with precision to the nearest tenth of a second. Six main categories of behavior were identified and used to label the video recordings: Breathing, Feeding, Gliding, Resting, Scratching and Swimming (Fig. 1). Any other observed behavior was categorized as Other. These seven categories are similar to those used to describe the behavior of green turtles in the previous studies in Martinique (see Jeantet et al., 2020a, for a precise description of the behaviors). The acceleration data, gyroscope data and pressure data associated with the corresponding observed behaviors were visualized and checked using R software (version 3.5.3; http://www.R-project.org/) and the *rblt* package (https://CRAN.R-project.org/package=rblt).

Data preprocessing mainly involved mapping the behavior labels to the multi-sensor data and removing the unlabeled night sequences for which the video recording was not available. No pre-processing was performed on the acceleration and gyroscopic data. For the pressure data, the difference was calculated between each data point (1 Hz) and a linear interpolation technique was then used to increase the sampling rate to 20 Hz.

In total, 69.7 h of multi-sensor recordings were labeled from six different hawksbill turtles (approximately 11.6 h of recording per individual, maximum 17.8 h, minimum 6.3 h, standard deviation 3.6 h). The predominant behavior observed in the videos was Feeding, totaling over 38.6 h, followed by Resting and Swimming, with 19.1 h and 7.9 h, respectively (Table S1). The other behaviors were expressed in minority (Breathing: 2.2 h, Gliding: 1 h, Scratching: 0.8 h and Other: 0.1 h).

### Datasets of the pre-trained models

#### Green turtle dataset

The green turtle dataset used in this study was collected in Martinique between February 2018 and May 2019 in a similar way to the hawksbill dataset. The CATS devices were deployed on 13 green turtles, *Chelonia mydas* (Linnaeus 1758), over a 1 or 2 day period. The dataset is annotated, freely available on the Dryad digital repository (https://doi.org/10.5061/dryad.hhmgqnkd9; Jeantet et al., 2020b) and has been precisely described in Jeantet et al. (2020a).

In total, the green turtle dataset contained 68.6 h of recordings from 13 individuals (approximately 5.29 h per individual, maximum 14.67 h, minimum 0.96 h, standard deviation 3.39 h), labeled with the same seven behaviors as the hawksbill dataset. The predominant behavior observed in the videos was Resting, totaling over 34.3 h, followed by Swimming and Breathing, with 22.3 h and 5.7 h, respectively (Table S1). The other behaviors were expressed in minority (Gliding: 2.3 h, Feeding: 1.8 h, Scratching: 1.2 h and Other: 1 h).

#### Human dataset

To evaluate the significance of the species used for pre-training, we also assessed the potential for transfer learning from other taxa, where data collection is more feasible. We focused on humans, as collecting data from them is significantly easier compared with other animals, resulting in numerous open-access datasets being available. In this case, we used the publicly available Intensive Care Unit (ICU) HAR dataset (Reyes-Ortiz et al., 2013), which is a dataset commonly used in the development of deep learning models for HAR (Ramanujam et al., 2021; Zhang et al., 2022).

The ICU HAR comprised 30 individuals wearing a smartphone on the waist recording 3D acceleration and 3D angular speed sampled at 50 Hz. Each person performed six activities (Walking, Walking_upstairs, Walking_downstairs, Sitting, Standing and Lying) for durations ranging from 1 to 2 min each, amounting to approximately 220 h of labeled sequences (see Table S1 for the specific duration available per activity).

#### Architecture of the deep learning model

In this transfer learning study, we tested V-Net (Jeantet et al., 2021), a model originally adapted to identify the underwater behaviors of green turtles in Martinique. The V-Net model has the advantage of generating an output with the same length as the input, allowing for predictions at each data point, or time step, from the recorded data. It can directly predict behaviors from raw accelerometer data without requiring preprocessing steps such as segmentation, filtering or the calculation of descriptive variables (Jeantet et al., 2020a). Moreover, the V-Net model was specifically designed to handle small and imbalanced datasets (Milletari et al., 2016). Beyond its application to green turtles in Martinique, the model has demonstrated satisfactory results in identifying terrestrial behaviors in green turtles in French Guiana (Jeantet et al., 2022) and prey capture events in chinstrap penguins (*Pygocelis antarctica*) in Antarctica (Schoombie et al., 2024). This evidence suggests its ability to generalize across species and contexts. For the human dataset, we tested a closely related model, U-Net, which shares a similar architecture and has been widely used in human studies (Meena and Sarawadekar, 2023; Zhang et al., 2019b).

**Fig. 2.**
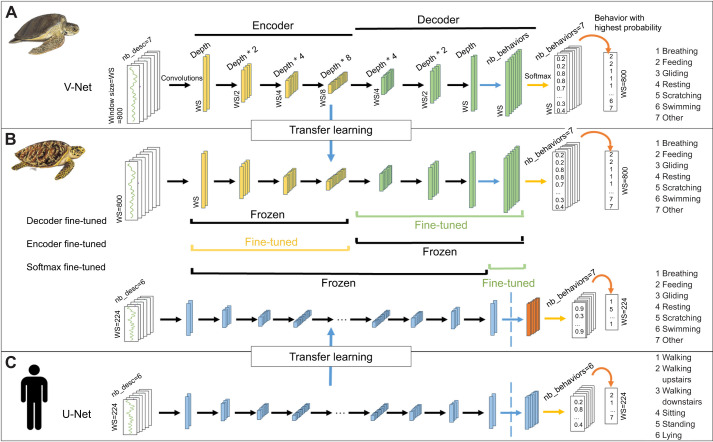
**Representation of the V-Net and U-Net architectures.** (A) The V-Net pre-trained on the green turtle dataset; (C) the U-Net pre-trained on the human dataset. (B) The transfer learning applications tested on the hawksbill dataset, highlighting the different frozen layer configurations for the model pre-trained on the green turtle dataset and the modified layer, shown in orange, for the model pre-trained on the human dataset. The black arrows represent two consecutive convolutional layers, while the blue arrows represent a single convolutional layer. Depth corresponds to the number of features to generate (depth=32), nb_desc refers to the number of input data, and nb_behaviors denotes the number of behaviors. WS represents the window size.

V-Net and U-Net are models also known as fully convolutional neural networks given that their primary operation is convolution. In machine learning, particularly in convolutional neural networks, convolution is a mathematical operation used to detect and extract hierarchical features from input data, such as edges, textures and patterns in images or signals. It operates by sliding a filter over an input, multiplying the filter values with the signal values, and summing them at each position to generate a new signal that highlights specific features based on the filter. The values of the filter involved in the multiplications and summations represent the model's weights. The training phase of a neural network optimizes these weights to enable accurate identification of the behaviors of the studied species based on the labeled data provided to the model. A convolutional layer applies a set number of filters to the input data. Each filter produces a transformed version of the input signal, referred to as a feature map. A fully convolutional neural network is characterized by the sequential arrangement of multiple convolutional layers (see Fig. 2 for a schematic visualization of the V-Net and U-Net architecture). The sequential application of multiple filters enables the extraction of various features from the signal, which subsequently enables behavior classification.

V-Net and U-Net are encoder–decoder architectures (Fig. 2). In these models, a series of convolutional layers first reduces the dimensionality of the input, mapping it to a lower-dimensional space. This process forms the encoder, and it generates a large number of discriminative features based on the number of layers and filters in the encoder. Following that, the second part of the model, known as the decoder, synthesizes these features to predict the outcome. This results in an output matrix with the same length as the input. The final layer is a convolutional layer with seven channels, each corresponding to one of the behavioral classes. At this stage, the outcome is a matrix containing a value for each behavior at every data point. These seven values are then passed through a softmax function (Eqn 1) to obtain the probability of each behavior for each data point.
(1)

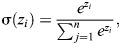
where σ is softmax and *z_i_* represents the *i*th element of the array of size *n*, where *n* is the number of classes. The softmax function produces an array of non-negative values that sum to 1, making it interpretable as a probability distribution. For each data point, the behavior with the highest probability is then selected as the predicted behavior. A detailed description of the V-Net and U-Net architectures is provided in the Supplementary Materials and Methods.

#### Evaluation of the models

To evaluate the performance of the various models employed in this study, we tested them on a dataset distinct from the training set. This approach enables us to assess the models’ ability to make predictions on data considered unseen as not used within model training. For the three datasets previously described, the data were divided into training, validation and testing sets. The training and validation sets are used simultaneously during the iterative training process, with the validation set employed to assess the performance trends. The testing set is reserved for the final analysis of the model, simulating the conditions under which the model will be applied.

For the human dataset, the dataset was originally provided in two datasets, with 70% of the individuals randomly allocated to the training set and the remaining 30% to the test set (Reyes-Ortiz et al., 2013). From the human training dataset, 30% of the individuals were randomly allocated to the validation dataset. For green turtles, seven individuals were used for training, three for the validation and three for testing, following the same distribution as in Jeantet et al. (2021). For the hawksbill turtles, three individuals were randomly selected for the training dataset, one for validation and two for testing. This data splitting approach was maintained consistently across all tested models.

We evaluated the model's generalizability using the testing dataset. To do so, we divided the entire recording for each test individual into consecutive windows with a 10% overlap. We ran the trained model on all windows and then reassembled the predictions to reconstruct the signal. Based on the predictions and the manually verified labels, we generated the confusion matrix and calculated the global accuracy (Eqn 2), precision (Eqn 3), recall (Eqn 4) and F1-score (Eqn 5):
(2)

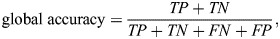

(3)

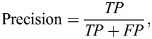

(4)

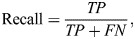

(5)

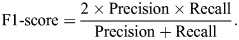
In these equations, TP (true positives) and TN (true negatives) refer to the number of correctly identified behaviors, while FN (false negatives) and FP (false positives) refer to the number of misclassified behaviors. For imbalanced datasets, the F1-score is considered a more reliable measure of model performance than the global accuracy (Saito and Rehmsmeier, 2015).

### Transfer learning

#### Fundamentals of transfer learning

As a neural network undergoes training, the model learns from annotated data and the weights are iteratively updated to minimize the difference between the outputs to the annotated values. Initially, these weights are randomly generated and then optimized for the specific classification task. Overfitting arises when these weights become overly tailored to the labeled dataset, leading to poor generalization and diminished model performance on new data (Ying, 2019). This issue is particularly prevalent when the model possesses a large number of weights and the training dataset's size is small.

Transfer learning presents a solution by leveraging pre-trained models, where the weights from a model *A* are employed to initialize model *B*. Typically, model *A* is trained on a similar task to model *B*, but with a larger dataset. In this process, the weights from model *A* are employed as the starting point for model *B*, as opposed to randomly initialized weights. During the training phase, the weights of model *B* are then fine-tuned specifically for task *B*, using the smaller labeled dataset. In practice, the initial layers of the model are often ‘frozen’, meaning their weights remain unchanged during training, while only the weights of the layers closer to the output are fine-tuned. Freezing certain layers offers computational advantages by reducing the number of weights that need to be optimized. The rationale for freezing the early layers of a model is that these layers capture general features of the data, while the deeper layers progressively learn more abstract representations (Gu et al., 2018; Yosinski et al., 2015 preprint). As a result, the deeper layers are more specialized for the specific task and must be adapted during transfer learning.

#### Pre-training on green turtle and human dataset

The first step of transfer learning involves training model *A* on the original dataset. To obtain the pre-trained model from the green turtle dataset, we trained the V-Net using the same parameters as in Jeantet et al. (2021). As input, we used the raw acceleration (*X*, *Y*, *Z*), the gyroscope (*X*, *Y*, *Z*) and the difference of pressure, sampled at 20 Hz over a window size of 40 s, resulting in a 7×800 matrix (Fig. 2). We employed the Generalized Dice loss (Sudre et al., 2017) and we trained the V-Net on 30 epochs with a batch size of 32, using the Adam optimizer and a learning rate of 0.0001. We obtained an F1-score of 81.1% and a global accuracy of 97.2% on the green turtle dataset.

For the human dataset, we employed the U-Net model proposed by Zhang et al. (2019b), which is publicly available on GitHub (https://github.com/zhangzhao156/Human-Activity-Recognition-Codes-Datasets). The U-Net model from Zhang et al. (2019a,b) consists of 23 convolutional layers, while the V-Net model used for the green turtle comprises 15 convolutional layers (Fig. 2, see Supplementary Materials and Methods for a detailed description of the V-Net and U-Net architectures). In their study, the authors evaluated the model on the ICU HAR dataset, using a fixed-length window of size 224 to segment the data. Following this approach, we trained the U-Net on the ICU HAR dataset using raw acceleration (*X*, *Y*, *Z*) and gyroscope (*X*, *Y*, *Z*) data over a 224-length window, resulting in a 6×224 matrix. The model was trained for 50 epochs with a batch size of 32, using the Adam optimizer, a cross-entropy loss and a learning rate of 0.001, consistent with Zhang et al. (2019a,b). We obtained an F1-score of 85.6% and a global accuracy of 95.7% on the human dataset.

#### Transfer learning on the hawksbill dataset

To assess the benefits of using transfer learning to identify the behavior of hawksbill turtles from a small dataset, we explored various scenarios. First, to determine whether a new model is necessary for identifying the behavior of hawksbill turtles, we predicted their behaviors using the V-Net trained on green turtles. In this case, no transfer learning was employed, and the model was not fine-tuned on the hawksbill dataset, but instead solely applied on the six individuals to assess the predictions. Second, we established a baseline model (Model-Hawksbill) by training the V-Net from randomly initialized weights on the hawksbill dataset. In this case, we did not use the pre-trained model on green turtles, but randomly initialized all the layers of the V-Net and trained it using the hawksbill dataset. Third, we used transfer learning on the V-Net pre-trained on the green turtle dataset (Model-Green_turtle). Finally, we used transfer learning on the U-Net pre-trained on the human dataset (Model-Human). We adapted the pre-trained U-Net architecture for our case study by replacing the final convolutional layer with a convolutional layer featuring seven channels (corresponding to the seven behavioral categories in the hawksbill dataset versus six in the human dataset) and fine-tuned all the layers (Fig. 2).

Additionally, as transfer learning involves freezing and fine-tuning different layers, we explored four additional scenarios for the Model-Green_turtle to determine the optimal configuration (Fig. 2). In the first scenario, we fine-tuned all the model layers. In the second, we froze the encoder weights and fine-tuned only the decoder weights. In the third scenario, we tested the opposite approach by freezing the decoder and fine-tuning the encoder weights. Finally, in the fourth scenario, we fine-tuned only the weights of the last convolutional layer.

To train or fine-tune the V-Net (Model-Hawksbill, Model-Green_turtle) on the hawksbill dataset, we fed the algorithm with a window size of 800 (40 s), encompassing data from the three accelerometer and three gyroscope axes along with the difference of pressure (matrix size 7×800). For Model-Human, we used a window of size 224 and only the three accelerometer and three gyroscope axes (matrix size 224×6), reproducing the input shape used with the human dataset. To generate these windows, we reused the method described in Jeantet et al. (2021), allowing us to produce a predetermined number of windows while fostering specific behaviors to balance the dataset (see Jeantet et al., 2021, for detailed information). We generated 6000 windows from the training dataset and 3000 from the validation dataset at each epoch (see Fig. S1 for the distribution of the behaviors based on this method). We ran each execution 20 times with the same hyperparameters (epoch=20, learning rate=0.0001, batch size=32, Adam optimizer, the Generalized Dice loss function for the V-Net and cross-entropy loss function for the U-Net). At each epoch, the model's performance was evaluated on the validation dataset, and throughout the 20 epochs, the model weights were saved when the best performance was achieved.

Models were implemented in Python3 using the Tensorflow2 and Keras libraries (Abadi et al., 2015 preprint; https://github.com/fchollet/keras). Model training and testing were performed on a Dell G15 with a 13th generation Intel Core i7-13650HX processor as Central Processing Unit (CPU) and an NVIDIA GeForce RTX 3050 Graphics Processing Unit (GPU). Pre-trained models and analysis scripts are available online on GitHub (https://github.com/AIMS-Research/research_za/tree/main/biologging_transferlearning_hawksbill), and the hawksbill dataset to run the scripts is available on Zenodo (https://doi.org/10.5281/zenodo.11402241).

## RESULTS AND DISCUSSION

**Fig. 3.**
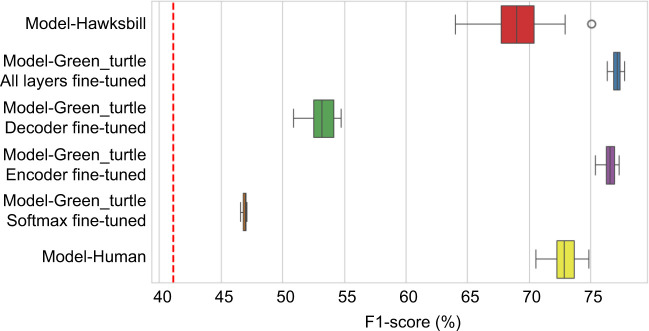
**Boxplots of the F1-scores obtained for each run across all experiments.** The dashed red line indicates the F1-score of the model trained on green turtles and applied to the hawksbill dataset without using transfer learning (F1-score=41.17%). The boxplot displays the median (central line), interquartile range (IQR, box boundaries) and data spread for *n*=20 runs. Whiskers represent variability outside the upper and lower quartiles, extending up to 1.5 times the IQR. Data points beyond this range are shown as outliers (dots).

**Fig. 4.**
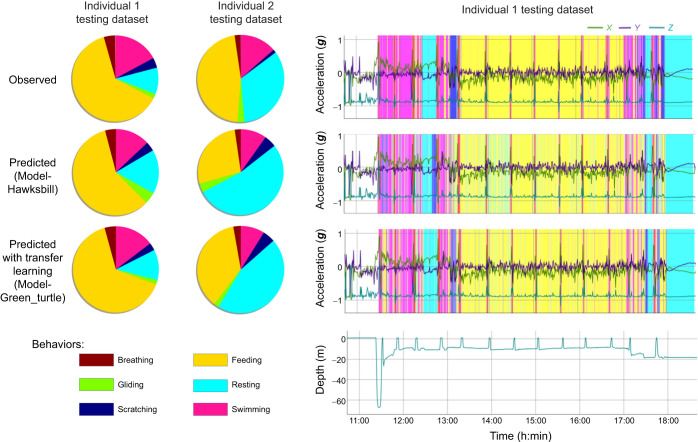
**Comparison of time budgets for two individuals from the testing dataset.** Observed behaviors from video analysis, predictions from a model with randomly initialized weights, and predictions from transfer learning using a model pre-trained on green turtles with all layers fine-tuned. The plots on the left show the time budget as a pie chart, while the plots on the right visualize the predictions over time for one individual, using the *rblt* package (https://CRAN.R-project.org/package=rblt).

In this study, we explored the use of transfer learning across species and taxa, employing fully convolutional neural networks to predict the behaviors of an endangered species and tackle the challenge of data scarcity. Initially, we found that while behaviors may seem similar between green and hawksbill turtles, a model trained solely on green turtles cannot accurately predict hawksbill behavior (Fig. 3; F1-score=41.17%), highlighting the necessity for species-specific model training. Conversely, utilizing a pre-trained model (Model-Green_turtle) significantly enhanced predictions, achieving an 8% point improvement compared with a model trained with randomly initialized weights (Fig. 3, Table 1; Model-Hawksbill, F1-score=69.11%; Model-Green_turtle with all layers fine-tuned, F1-score=77.12%). Comparing the time budget predicted for the testing dataset from the Model-Hawksbill and the Model-Green_turtle with camera observations, the predictions from the Model-Green_turtle show a closer alignment with the observed data for both individuals (Fig. 4). The time budget, representing the proportion of time allocated to each behavior, was calculated by dividing the number of predicted data points for each behavior by the total number of labeled data points. For both individuals, particularly individual 2, a closer inspection of the predictions revealed Feeding sequences that were predicted as Resting (Fig. S2). These Feeding sequences correspond to accelerometer and gyroscope signals with minimal variation, probably reflecting moments when the turtle was chewing its food. Although these chewing sequences were labeled as Feeding based on the video, it was difficult to observe the actual chewing, as the turtle typically had its head down. Consequently, some errors may result from our annotation of the accelerometer data – either when the turtle is not actually chewing or when the chewing is not detected by the logger – rather than from a model error. Despite being trained on only three individuals, the model demonstrates high accuracy in behavior identification and shows promising potential for broader applications.

**Table 1.**
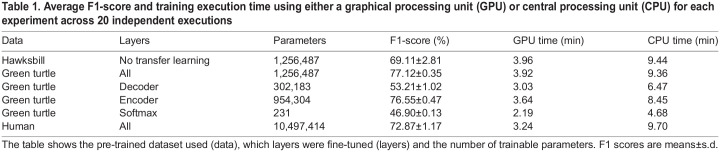
Average F1-score and training execution time using either a graphical processing unit (GPU) or central processing unit (CPU) for each experiment across 20 independent executions

This study presents the first application of transfer learning for wildlife monitoring using acceleration data. To our knowledge, only two studies have used transfer learning for acceleration-based behavior identification in livestock. Kleanthous et al. (2022a) evaluated the effectiveness of transfer learning in identifying sheep behavior using two datasets collected simultaneously from accelerometers positioned differently on the collars of the same individuals. Bloch et al. (2023) applied transfer learning to study cow behavior, using a pre-trained model from an open-source dataset of different cows in a different environment. Likewise, a review on transfer learning applied to HAR revealed that in most studies, models are pre-trained either on the same individuals with sensors positioned at different locations or on different subjects with loggers in the same location and/or in different environments (Hernandez et al., 2020). In these studies, and across the literature, cross-species transfer learning has not been attempted. In our case, as similar datasets for hawksbills are not available, we demonstrate that transfer learning can also be applied across different species. We show that predictive accuracy improves when the model is trained on species with similar behaviors and environment, but also when trained on species with entirely different behaviors such as humans (3.8% points increase in the F1-score, Model-Hawksbill, F1-score=69.11%; Model-Human, F1-score=72.87%; Table 1).

Transfer learning from human data yields better results than a model trained from randomly initialized weights, suggesting that the species used for pre-training can be entirely different from the species under study while still benefiting the model. This occurs because deep learning models consist of multiple successive layers, with each layer progressively representing data in increasingly abstract forms from the input to deeper layers (Goodfellow, 2016). As the model has already been trained to recognize human behaviors, its weights are optimized to extract signal features that distinguish between behaviors, with the earlier layers of the encoder capturing general features and the deeper layers identifying finer, more detailed aspects of the signal (LeCun et al., 2015; Zeiler and Fergus, 2014). Although these finer features differ between turtle and human behaviors, the fine-tuning phase enables it to adapt and optimize its weights to capture fine-grained features of the new species. In their survey on transfer learning, Weiss et al. (2016) draw an analogy, suggesting that a person with guitar-playing experience and a musical background is likely to learn to play the piano more quickly than an individual lacking prior musical experience. These findings are particularly promising for the broader application of deep learning and transfer learning in acceleration-based behavior identification for wildlife monitoring, independent of the species under study. The results presented in this study suggest that the species used to pre-train the model can differ from the species being studied, yet this approach can still produce better outcomes than a model trained solely on the available data. From an applied perspective, practitioners using accelerometer data can use a model pre-trained on human data as a starting point for developing species-specific models. This could help reduce the amount of data needed for training models, which is a significant advantage for species that are difficult to observe in their natural habitats.

The primary risk of training a deep learning model on a small dataset is overfitting. The significance of a machine learning model's ability to generalize lies in its capacity to effectively handle variations in data. A well-generalized model avoids learning noise or overly specific patterns from the training data, instead capturing the essential, generalizable features that are critical for accurately recognizing relevant behaviors across diverse datasets or individuals. However, achieving good generalization is particularly challenging when the training dataset is small, as the model is more prone to overfitting to the limited data available (Ying, 2019). In our study, we tested the model on a set of new, unseen individuals that were not included in the training data to assess its generalization performance. The observed improvement in the F1-score following the application of transfer learning suggests that the model has enhanced its ability to generalize to new data. In addition, a common method to evaluate overfitting is to visualize the model's performance on both the training and validation datasets during training. If the model performs well on the training data but poorly on the validation data, it indicates overfitting. In this study, the visualization of performance trends during training for both a randomly initialized model and a model using transfer learning suggests that the transfer learning model exhibited less overfitting (Fig. S3). Training the model on a larger dataset allows it to learn from a broader range of examples, enabling it to capture more diverse patterns and variability among individuals. This helps the model identify key features that differentiate behaviors in a way that is independent of specific individuals, thus enhancing its ability to generalize. The model's ability to avoid overfitting and generalize effectively is promising for its application to new, unseen individuals, suggesting its robustness and potential for real-world applications.

Another advantage of transfer learning highlighted in this study is the reduced variation in the F1-scores across repeated independent executions, indicating a more robust model with transfer learning (Model-Hawksbill, s.d.=2.81%; Model-Green_turtle with all layers fine-tuned, s.d.=0.35%; Table 1). Even using transfer learning with human data reduced this variability (Model-Human, s.d.=1.17%). Our study shows that the model performance can vary by more than 10% points when starting from a random initialization of weights (Fig. 3). This is probably because the initial values of the weights affect the ability to achieve the suitable weights during training. Depending on the initial weights, training may not reach suitable values, resulting in lower performance in some cases. Another explanation could be that the model is less sensitive to individual variations and more consistent in its ability to predict behavior for new, unseen individuals. From an applied perspective, having a robust model is particularly important as, in most cases, the model is trained only once before being applied to new data.

Deep learning remains relatively underutilized in wildlife monitoring based on acceleration data, possibly because deep learning models are often considered black boxes with a difficulty in identifying the information used by the models (Castelvecchi, 2016). In this study, we aimed to enhance our comprehension of transfer learning and deep learning models by fine-tuning different layers and evaluating the outcomes (Fig. 3, Table 1). We demonstrate that, with an encoder decoder network, better results are attained when fine-tuning either the entire model (F1-score: 77.12%) or the first layers, the encoder (F1-score=76.55%), highlighting the significance of these early layers in adapting the model to a specific task or, in this case, a specific species. The weaker results observed when the encoder is ‘frozen’ corroborate this finding (F1-score=53.21%). In transfer learning applications within computer vision and bioacoustics, it is typically the final layers of the pre-trained model that undergo fine-tuning (Iman et al., 2023). In our study, the encoder emerges as the pivotal component that requires customization to suit the specific task or species under study. Based on our results, we can speculate that the decoder, responsible for signal reconstruction, is less species specific as we obtained comparable results whether the decoder was fine-tuned along with the encoder or maintained ‘frozen’. These results also suggest a low dependency between the encoder, responsible for extracting information, and the decoder. Lastly, while models in computer vision or bioacoustics yield favorable results with softmax fine-tuning alone (Batist et al., 2024; Dufourq et al., 2022), this approach yielded remarkably poor results in our study (F1-score=46.90%). These results provide a clearer insight into the layers of the V-Net responsible for acquiring problem-specific knowledge. While transfer learning has become a commonly used approach in both bioacoustics and computer vision, we demonstrate that its implementation can vary significantly depending on the model architecture and the specific classification task involved. Although our study primarily focuses on developing a robust method for wildlife monitoring, our findings also enhance comprehension of the implicated models, thereby offering potential benefits to the wider deep learning and ecology communities.

One of the primary advantages of freezing layers in deep learning models is the reduction of computational cost during training. In the context of transfer learning, only the weights of unfrozen layers are fine-tuned, reducing the number of calculations required to optimize the model. For example, in bioacoustics, researchers have used the pre-trained model ResNet152V2, with 58,331,648 weights, and have fine-tuned only the softmax layers with 81,922 weights (Batist et al., 2024). As a result, with fewer weights to optimize, the training time is significantly reduced. In our case, achieving the best results requires either fine-tuning all the weights, which does not reduce training time compared with a model with randomly initialized weights (3.92 min with GPU for the Model-Green_turtle versus 3.96 min for the Model-Hawksbill), or fine-tuning only the encoder. In the latter scenario, we observed only a slight improvement in training time, with a reduction of 59 s without a CPU and 19 s with a GPU compared with the Model-Hawksbill (Table 1). The reduction in training time becomes more significant when only the decoder or softmax layer is fine-tuned, but this comes with a decrease in performance. Nevertheless, while we did not observe a significant improvement in training times due to transfer learning, the training durations remain reasonable, averaging around 10 min without a GPU.

In conclusion, we demonstrate that transfer learning improves behavior classification using accelerometer data of an endangered species, particularly in situations where datasets are scarce. By leveraging transfer learning, we developed a more robust model with reduced variability in predictive performance and better generalization, evidenced by a higher F1 score on new individuals compared with a model trained from randomly initialized weights. We also demonstrate that transfer learning can be successfully applied using data from entirely different species – such as humans and hawksbill turtles – though better results are obtained when the species share similar morphology, as seen with the green turtle and hawksbill turtle. From a practical perspective, it remains essential to collect data and label these date through direct observations, which can be particularly challenging for endangered species that are difficult to monitor. Nevertheless, the amount of labeled data required for training can be significantly reduced through transfer learning. While estimating the minimum number of individuals needed is difficult because of task-specific issues – such as the difficulty of distinguishing the associated accelerometer signals – transfer learning generally decreases the number of individuals required. It is therefore feasible to pre-train models on larger datasets available online that may be similar to the species of interest or on human data when specific data are lacking. As transfer learning can be applied to all deep learning architectures, it is also possible to reuse existing models available online, usually provided with the data they were trained on (Jeantet et al., 2021; Otsuka et al., 2024; Schoombie et al., 2024; Zhang et al., 2019b). Fine-tuning can be applied to all layers, particularly in models with relatively few parameters, or to specific layers to adapt the model to the species under study. We demonstrate that the choice of layers for fine-tuning may vary depending on the architecture, highlighting the need for a review of common practices from other fields according to the chosen model architecture. Therefore, transfer learning serves as a valuable resource for researchers, enabling them to adapt existing models to their study species and saving time by eliminating the need to develop species-tailored models, which is a common practice. In this context, it is crucial for practitioners to share their accelerometer data and associated models, allowing the broader community to benefit and ultimately facilitate the utilization of accelerometers for wildlife monitoring and expanding its scope of application.

## Supplementary Material

10.1242/jexbio.249232_sup1 Supplementary information
