# Supplementary material for: Automatic identification of the endangered hawksbill sea turtle behavior using deep learning and cross-species transfer learning
Source: J Exp Biol. 2024 Dec 23;227(24):jeb249232. doi: 10.1242/jeb.249232 (PMC11698059; doi:10.1242/jeb.249232)
Supplement: Supplementary information [file jexbio-227-249232-s1.pdf]

Supplementary Materials and Methods

A Appendix

Architecture of the V-Net and the U-Net

The U-Net was introduced by Ronneberger, Fischer, and Brox (2015) for detecting organs of interest in 2D biomedical images. The V-Net, introduced by Milletari, Navab, and Ahmadi (2016), is an adaptation of the U-Net designed for 3D biomedical images. Both share the same architecture with an encoder and decoder and the novelty of the V-Net lies in its use of 3D convolutions instead of 2D. Both models consist of layers, where each layer includes two convolutional layers followed by downsampling in the encoder or upsampling in the decoder. Another innovation of the V-Net lies in the addition of a "shortcut," where the input of the first convolution is added to the output of the second convolution at each layer. Milletari et al. (2016).

The V-Net developed on the green turtle dataset is therefore a fully convolutional network, with successive sequence of 1D convolutions based on the time dimension (Jeantet, Vigon, Geiger, & Chevallier, 2021). Each convolutional layer is followed by a ReLU activation. Downsampling is achieved by performing a convolution with a kernel size of 2 and a stride of 2. Upsampling is achieved by doubling the data size, effectively creating a copy of the data along the time dimension. The model consists of 3 layers corresponding to 8 convolutional layers and 3 downsampling operations in the encoder, as well as 6 convolutional layers and 3 upsampling operations in the decoder. The last layer is a 1D convolution with softmax activation to obtain the probability for each behavior.

The U-Net used in Zhang et al. (2019) is similar to the one proposed by Ronneberger et al. (2015), with the addition of a sequence of double convolutions. The model comprises 5 layers, consisting of 12 convolutional layers and 5 downsampling operations in the encoder, along with 10 convolutional layers and 5 upsampling operations in the decoder. Downsampling is performed using max pooling. In Zhang et al. (2019), the authors applied 2D convolutions to acceleration-gyroscopic time series data. The final layer is a 2D convolution with a softmax activation function, which produces probabilities for each behavior.

References

Jeantet, L., Vigon, V., Geiger, S., & Chevallier, D. (2021). Fully convolutional neural network : a solution to infer animal behaviours from multi-sensor data. *Ecological Modelling*, *450*(109555). doi: 10.1016/j.ecolmodel.2021.109555

Journal of Experimental Biology • Supplementary information

Milletari, F., Navab, N., & Ahmadi, S. A. (2016). *V-net: Fully convolutional neural networks for volumetric medical image segmentation* (No. arXiv:1606.04797v1). arXiv. (ISBN: 9781509054077) doi: 10.1109/3DV.2016.79

Ronneberger, O., Fischer, P., & Brox, T. (2015). *U-net: Convolutional networks for biomedical image segmentation* (No. arXiv:1505.04597). arXiv. Retrieved 2024-04-24, from `http://arxiv.org/abs/1505.04597` doi: 10.48550/arXiv.1505.04597

Zhang, Y., Zhang, Z., Zhang, Y., Bao, J., Zhang, Y., & Deng, H. (2019). Human activity recognition based on motion sensor using u-net. *IEEE Access*, *7*, 75213-75226. doi: 10.1109/ACCESS.2019.2920969

Journal of Experimental Biology • Supplementary information

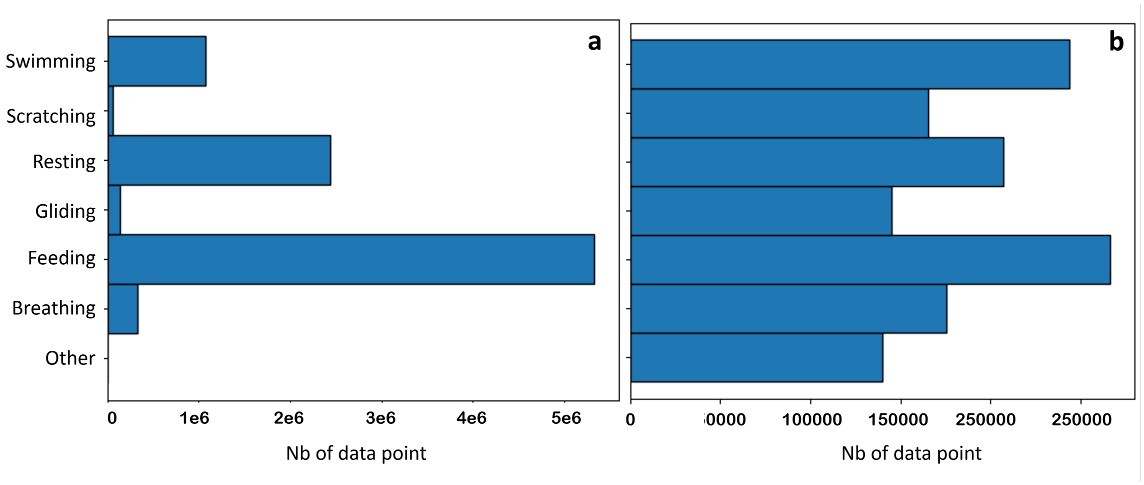

Fig. S1. Histogram of the distribution of behaviors in the hawksbill training dataset when using a sliding window (a) or the global generator described in Jeantet et al. (2021) (b). Figure (b) shows the number of data points for each behavior when 6000 windows of size 800 (40 sec) are drawn. Figure (a) represents the number of data for each behavior when using a sliding window of size 800 on the entire training data with an overlap of 200 (10 sec).

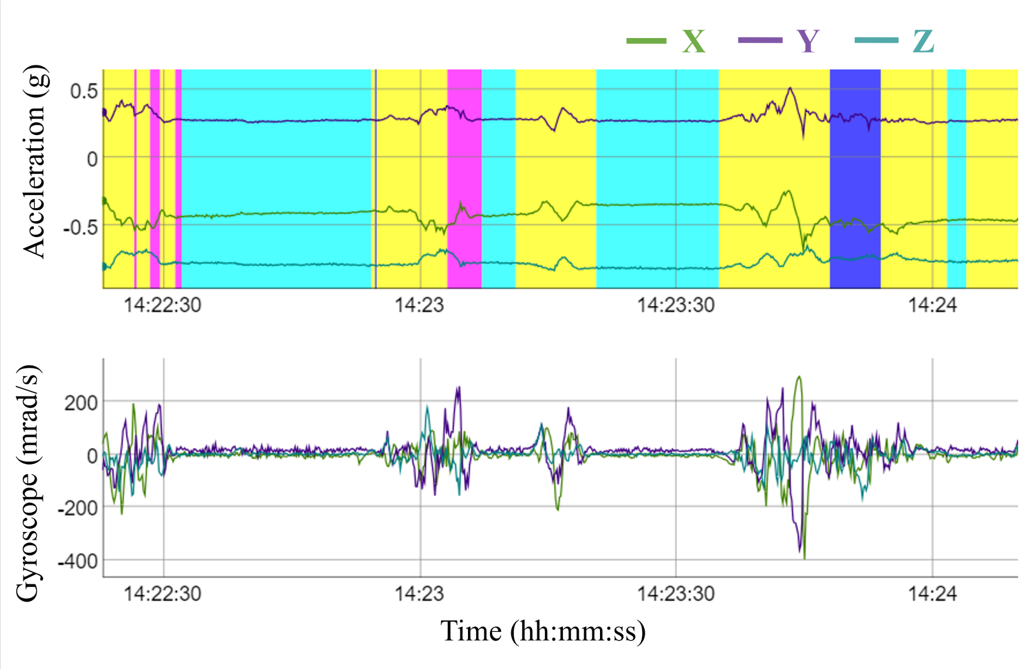

Fig. S2. Predictions from a model pre-trained on the green turtle dataset with all its layers fine-tuned on the hawksbill dataset for a sequence labelled as Feeding of a hawksbill turtle from the testing dataset (individual #2)."

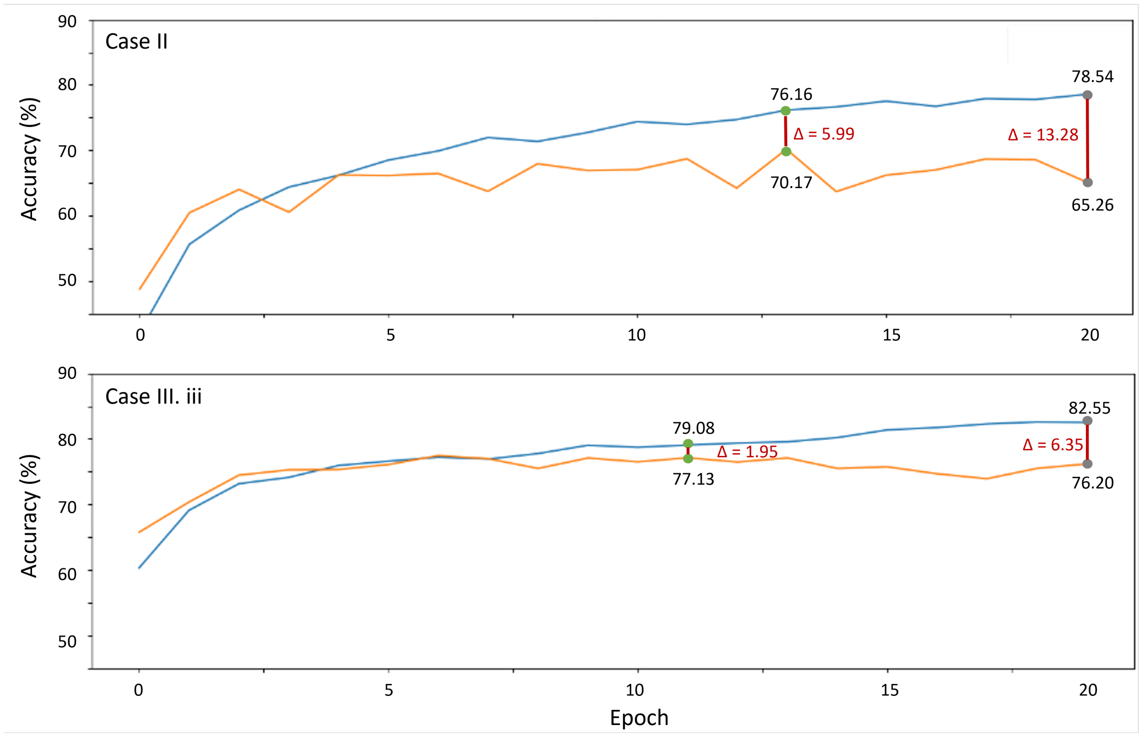

Fig. S3. Accuracy at each epoch on training and validation Hawksbill datasets: comparison between a V-Net with randomly initialized weights and a pre-trained V-Net on green turtle dataset with frozen decoder. The green dots represent the epoch at which the model was saved and the black dots the last epoch. A significant difference between the validation and training data indicates that the model cannot generalize to new data, suggesting that the weights are overly adjusted to the training dataset (overfitting).

Table S1. Presentation of the datasets used in the study, showing the different behaviors and the corresponding recording time available.

	**Hawksbill turtle dataset**	**Green turtle dataset**		**Human dataset**
Number of individuals	6	13		30
Breathing	2.2 h	5.7 h	Walking downstairs	29.9 min
Feeding	38.6 h	1.8 h	Standing	40.6 min
Gliding	1.0 h	2.3 h	Sitting	38.6 min
Resting	19.1 h	34.3 h	Walking	36.9 min
Scratching	0.8 h	1.2 h	Laying	41.2 min
Swimming	7.9 h	22.3 h	Walking upstairs	33.0 min
Other	0.1 h	1.0 h		

Journal of Experimental Biology • Supplementary information
